# Gastrointestinal Tolerance of D-Allulose in Healthy and Young Adults. A Non-Randomized Controlled Trial

**DOI:** 10.3390/nu10122010

**Published:** 2018-12-19

**Authors:** Youngji Han, Bo Ra Choi, So Young Kim, Seong-Bo Kim, Yang Hee Kim, Eun-Young Kwon, Myung-Sook Choi

**Affiliations:** 1 Department of Food Science and Nutrition, Kyungpook National University, 1370 San-Kyuk Dong Puk-Ku, Daegu 41566, Korea; youngji.kor.han@gmail.com (Y.H.); borachoi15@naver.com (B.R.C.); ksd1372@hanmail.net (S.Y.K.); savage20@naver.com (E.-Y.K.); 2 Center for Food and Nutritional Genomics Research, Kyungpook National University, 1370 San-Kyuk Dong Puk-Ku, Daegu 41566, Korea; 3 Food R&D, CJ CheilJedang Corp., Seoul 04560, Korea; seongbo.kim@cj.net (S.-B.K.); yanghee.kim@cj.net (Y.H.K.)

**Keywords:** allulose, monosaccharide, sugar substitute, rare sugar, gastrointestinal tolerance test

## Abstract

D-allulose has recently received attention as a sugar substitute. However, there are currently no reports regarding its association with gastrointestinal (GI) tolerance. Thus, we performed a GI tolerance test for D-allulose in order to establish its daily acceptable intake level. When the dose of D-allulose was gradually increased in steps of 0.1 g/kg·Body Weight (BW) to identify the maximum single dose for occasional ingestion, no cases of severe diarrhea or GI symptoms were noted until a dose of 0.4 g/kg·BW was reached. Severe symptoms of diarrhea were noted at a dose of 0.5 g/kg·BW. Similarly, the GI tolerance test did not show any incidences of severe diarrhea or GI symptoms until a dose of 0.5 g/kg·BW was reached. A correlation analysis of the GI tolerance test for D-allulose and sugar revealed significantly higher frequencies of symptoms of diarrhea (*p* = 0.004), abdominal distention (*p* = 0.039), and abdominal pain (*p* = 0.031) after D-allulose intake. Increasing the total daily D-allulose intake gradually to 1.0 g/kg·BW for regular ingestion resulted in incidences of severe nausea, abdominal pain, headache, anorexia, and diarrheal symptoms. Based on these results, we suggest a maximum single dose and maximum total daily intake of D-Allulose of 0.4 g/kg·BW and 0.9 g/kg·BW, respectively.

## 1. Introduction

Rapid recent advancements have contributed to an increased human lifespan. However, a significant proportion of adults have metabolic diseases caused by fundamental problems related to their diets and lifestyles, making healthcare and treatment a social issue [1]. In particular, modernized diets and lifestyle changes have led to an increased number of patients with metabolic syndrome [2]. The excessive consumption of carbohydrates ultimately leads to their storage as triglycerides in the body [3,4]. In addition, the intake of surplus energy causes overweightness and obesity, resulting in elevated blood glucose levels and hyperlipidemia as a complication of obesity [5].

However, the use of sugar as a primary sweetener is on the decline in modern society, owing to the desire of consumers to pursue healthy lifestyles [6,7]. Thus, the focus has recently shifted to the development of economical and safe sweeteners to replace sugar [8,9]. Low- or zero-calorie sugar substitutes that have a sweet taste are preferred. Allulose, a C-3 epimer of D-fructose, is a rare sugar that has recently attracted significant interest [10]. D-allulose is a monosaccharide that is low in calories and that maintains approximately 70% of sweetness relative to sugar [11]. Dietary allulose transport is likely mediated by GLUT5 in the small intestine, and its affinity for GLUT5 is lower than that of fructose [12]. Previous studies have reported that allulose has properties that are similar to those of fructose, and that it also exhibits various biological functions [13,14,15,16]. A few clinical studies of the efficacy of allulose have been conducted [17,18]; however, there no report has described the GI capacity of D-allulose in the human population.

Ascertaining the acceptable daily intake level is essential in the application of new food substances in the food industry; for this purpose, gastrointestinal (GI) tolerance tests are performed [19]. These tests are conducted using groups divided by age, gender, and race, have been conducted to validate the use of sugar substitutes, including erythritol and xylitol, which have already been distributed in the market [20,21]. Therefore, it is necessary to establish the acceptable daily intake of D-allulose in the human population through the use of the GI tolerance test. Thus, we performed this test on a population of Koreans.

## 2. Materials and Methods

### 2.1. Subjects

Thirty healthy, normal range of body mass index (BMI) based on Asian criteria (18.5 kg/m^2^ ≤ BMI < 23 kg/m^2^), non-adapted volunteers (15 men and 15 women) aged between 21 and 30 years were recruited through an advertisement. The exclusion criteria were as follows:

(1) Hypertensive taking diuretic
(2) Patient taking oral hypoglycemic agent or insulin injection
(3) Serious cardiac, renal, hepatic, thyroid or cerebrovascular disease
(4) Serious cystic or gastrointestinal disease, gout or porphyria
(5) Psychiatric problems such as depressive disorder, schizophrenia, alcoholism, drug intoxication
(6) Using functional food products that may affect the results of this study
(7) A history of surgery within 6 months
(8) Cancer diagnosis and treatment
(9) Asthma or other allergy
(10) A history of surgery within the past 6 months
(11) Drinking frequently
(12) Anticipated difficulties following the taking experimental material schedule
(13) Pregnant or currently lactating

The purpose and nature of the proposed studies were explained to the volunteers, who then provided written consent. The volunteers were given the option to discontinue their participation in the study at any time and for any reason. The studies were approved by the Institutional Review Board of the Bioethics Committee of Kyungpook National University (KNU 2016-110) and the Clinical Research Information Service (CRIS) of Ministry of Health and Welfare of the Republic of Korea (KCT0002216). The subjects’ travel expenses were reimbursed, and they received remuneration to compensate for the inconvenience of participating in the study. The sample size was estimated using G·Power 3.1.9.2 (Heinrich-Heine-University of Dusseldorf, Dusseldorf, Germany). Assuming a statistical power of 90% and a significance level of 0.05, it was estimated that at least 18 participants would be needed to show a statistically-significant difference.

### 2.2. Test Materials

The test materials included 200 mL samples of green-colored, grape-flavored (1310371, Aroma Line, Korea), non-carbonated drinks containing either sucrose or allulose. All test materials were supplied by CheilJedang, Inc. (Seoul, Korea).

### 2.3. Experimental Design

**Figure 1:**
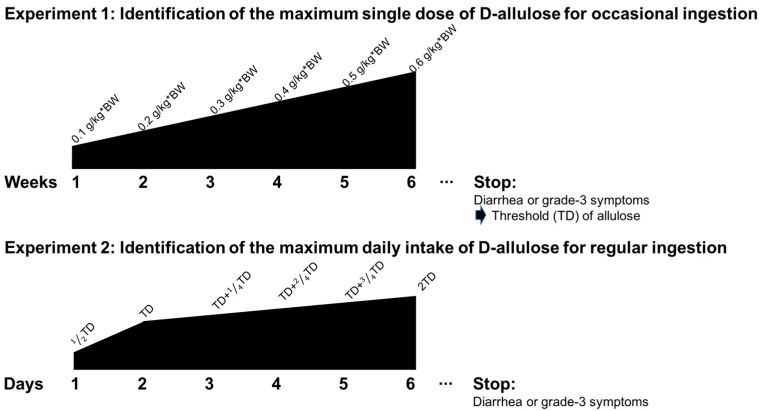
Experimental design of the D-allulose tolerance test to determine the maximum single dose and daily maximum intake.

Two separate experiments were performed as a single-group open study. Tolerance to D-allulose was assessed over two experiments, Experiments 1 and 2, separated by a gap of seven days. Each experiment corresponded to a different pattern of D-allulose consumption (Figure 1).

### 2.4. Thresholds

Experiment 1 involved the ingestion of allulose in order to determine the single maximum dose for occasional consumption. A daily single dose of D-allulose was administered for each week, starting with 0.1 g/kg·body weight (BW) for the first week, 0.2 g/kg·BW for the third week, 0.3 g/kg·BW for the fifth week, 0.4 g/kg·BW for the seventh week, 0.5 g/kg·BW for the ninth week, and 0.6 g/kg·BW for the 11th week. A wash-out period of one week was allowed every other week to avoid adaption. One female subject failed to complete Experiment 1 due to illnesses unrelated to the experiment. All male subjects completed the study satisfactorily. The amount of D-allulose consumed in the liquid beverage was calculated based on the unit body weight of the subjects; details are listed in Table 1. According to the rules set by the study, the GI test for D-allulose intake would be terminated if and when even one item in the diarrhea or GI symptom list was recorded at the severe level (grade 3) during the study period. Accordingly, the dose administered just before the termination of the study was defined as the maximum single dose of D-allulose for occasional ingestion.

After the maximum dose of D-allulose for occasional ingestion was identified in Experiment 1, the maximum total daily intake of D-allulose for regular ingestion was investigated in Experiment 2. Nineteen subjects (10 male and 9 female) voluntarily participated in the GI tolerance test for regular ingestion of D-allulose. The amount of D-allulose in the test beverages was determined based on the unit body weight of each subject (Table 2). The scheduled doses of D-allulose administered for each time period are shown in Table 3. The individuals who participated in Experiment 2 visited the study center every day, without wash-out periods. As in Experiment 1, the study was terminated if and when grade 3 diarrhea or GI symptoms were reported. Accordingly, the dose administered right before the termination was regarded as the maximum daily total intake of D-allulose for regular ingestion.

### 2.5. GI Symptoms

The subjects were provided printed sheets on which they were asked to record the incidences and magnitudes of the GI responses for the 24 h period following the consumption of the test products. The notable responses included nausea, bloating, borborygmi, colic, flatulence, headache, satiety, appetite diminution, and diarrhea. Each response was ranked on a hedonic scale, where 0 indicated ‘normal’ function, 1 indicated ‘a slightly higher number of symptoms than usual’, 2 indicated ‘a noticeably higher number of symptoms than usual’, and 3 indicated ‘a considerably higher number of symptoms than usual’.

### 2.6. Diet

Subjects were provided with a meal before allulose consumption. The constituents of the provided meal are shown in Table 4.

### 2.7. Statistics

The symptom responses were classified as categorical and were considered to be non-parametric. The GI responses following the consumption of the different test drinks were compared by 2 × 2 contingency table analysis (*x*^2^), as described by McNemar [22]. Binomial tests were used when the expected frequency in each cell of the contingency table was less than 5, while *x*^2^ tests were used to estimate the differences in the occurrences of multiple symptoms following the consumption of the test products. The symptom scores, following the consumption of the test drinks, were derived by the summation of each subject’s GI responses from 0 to 3, as described above.

## 3. Results

The characteristics of the individuals who participated in Experiment 1 are shown in Table 5. Sugar and D-allulose (initial dose: 0.1 g/kg·BW) were administered in doses that were gradually increased in steps of 0.1 g/kg·BW. The GI symptoms of the subjects, including diarrhea, were recorded after 24 h of administering D-allulose or sucrose for occasional ingestion, as shown in Table 6 and Table 7. D-allulose ingestion was gradually increased by 0.1 g/kg·BW. When 0.5 g/kg·BW of D-allulose was administered, 13 subjects (44.83%) reported diarrhea and four (13.79%) had severe symptoms. The GI tolerance for the occasional ingestion of sugar was compared to those for D-allulose, based on the dose. The symptom frequencies of diarrhea (*p* = 0.004), abdominal distention (*p* = 0.039), and abdominal pain (*p* = 0.031) were significantly higher when 0.5 g/kg·BW of D-allulose was administered compared to the same dose of sugar. Thus, the maximum single dose for the occasional ingestion of D-allulose was 0.4 g/kg·BW (about 25.43 g); this indicates that an individual with a body weight of 60 kg can consume a maximum of 24 g of D-allulose.

The characteristics of the individuals who participated in Experiment 2 to determine the maximum daily total intake of D-allulose as regular ingestion are shown in Table 8. The test results for the calculation of the maximum total daily intake of D-allulose are shown in Table 9. On the eighth day, when 1.0 g/kg·BW of D-allulose was administered, the subjects displayed severe levels of nausea (one subject), abdominal pain (one subject), headache (one subject), anorexia (one subject), and diarrhea (two subjects), as shown in Table 9. Thus, the maximum total daily intake of D-allulose was 0.9 g/kg·BW (57.80 ± 9.54 g), indicating that adults weighing 60 kg can consume a maximum of 54 g of D-allulose, daily.

## 4. Discussion

The excessive consumption of sugar and sugar-sweetened foods and drinks is associated with obesity, as it supplies a large number of calories with little or no nutritional value [23]. According to the Korea National Health and Nutrition Examination Survey (KNHANES), the mean total sugar intake of Koreans was 61.4 g/person/day, corresponding to 12.8% of the total daily energy intake [24]. More than half of this amount (35.0 g/day, 7.1% of the daily energy intake) was obtained from processed foods. The top five processed food sources for Koreans, in terms of sugar intake, were granulated sugar (4.9 g/day), carbonated beverages (3.5 g/day), coffee (3.3 g/day), breads (3.2 g/day), and fruit and vegetable drinks (2.1 g/day). Compared to other age groups, the total sugar intake of adolescents and young adults was much higher (69.6 vs. 68.4 g/day for 12–18 years and 19–29 years, respectively), with beverage-driven sugar intake amounting to up to 25% of the total intake. In addition, multiple nutrition surveys, such as the National Health and Nutrition Examination Survey (NHANES), have been undertaken in many countries. Based on these surveys, people in Australia and Ireland have total sugar consumptions of 103 and 92 g/day, respectively. People in Norway, Denmark, Ireland, and America consume 42, 48, 92, and 55 g/day of added sugars, respectively [25].

Reducing the intake of free sugars to less than 10% of the total daily energy intake was first recommended by the World Health Organization (WHO) Study Group in 1989 and was further elaborated by a joint WHO/ Food and Agriculture Organization of the United Nations (FAO) Expert Consultation in 2002 [26,27]. In 2015, the updated WHO guidelines called for a further reduction in the intake of free sugars to <5% of the total intake [28]. However, it is not a simple task to maintain this level of dietary sugar, as sugar consumption is very closely related to personal food preferences. Thus, it is a major challenge to develop low-calorie foods or drinks that are also tasty. The sweetness of sugar substitutes is generally much higher than that of sugar; thus, the addition of a smaller quantity of substitutes is sufficient to provide the same level of sweetness [29]. Some sugar substitutes are low in calories, while others such as erythritol have zero calories.

Allulose can be produced by epimerization of D-fructose using the enzymatic method of construction [30]. Other artificial sweeteners that contain almost no calories, such as aspartame, sucralose, saccharin, and cyclamate, have been developed, but their function is only to provide sweetness. However, allulose could lead to the normalization of body weight through the reduction of body fat mass in cases of diet-induced obesity under pair fed conditions for iso-caloric diets [26]. Allulose suppressed the absorption of dietary lipids and increased body fat oxidation [31]. In our previous clinical study (CRiS, KCT0002084), which was completely different to the present one, allulose reduced body fat mass compared to suclalose in overweight and obese subjects [18]. So, allulose can exert its biological effects regardless of its zero-energy density.

It is convenient for the food industry to use D-allulose, as its chemical properties are similar to those of fructose in terms of solubility, water activity, and viscosity. Accordingly, conducting a GI tolerance test for D-allulose is essential for its application in the food industry.

Several limitations of this clinical study should be emphasized. It is difficult to represent the general population in this small sample size. Also, we recruited young subjects who were within the normal range of BMI; considerably more symptoms may appear in individuals who are old, obese, or with certain metabolic and GI diseases, when they take 0.4 g/kg·BW or less allulose. Furthermore, since subjects had only a one-week wash-out period in the occasional ingestion part of the experiment, that may not be enough for the GI adaption with allulose. If we had used wash-out period of a couple of weeks, GI tolerance might be increased.

The results of the present study demonstrate that the GI response to D-allulose, in occasional or regular consumption in increasing doses, includes abdominal distention, abdominal pain, and diarrhea. Diarrhea was most common reason for subject withdrawal from the study. Tolerance to slowly-absorbed, bulking sweeteners is commonly evaluated through the participation of healthy volunteers who consume large or increasing amounts of the test substance by itself, diluted in water. However, individuals do not usually ingest a large load of such sugar-free products at one time, in a state of fasting; rather, they consume smaller amounts through the day, during or after meals, in the form of pastries or other confectionery items. In addition, individuals may consume slowly-absorbing bulk sweeteners occasionally or regularly.

## 5. Conclusions

In conclusion, a major aim of our study was to compare the digestive tolerance associated with D-allulose consumption throughout the day, in the aforementioned two manners. We identified the maximum single dose of D-allulose to be 0.4 g allulose/kg·BW for occasional ingestion and the maximum total daily intake for this sugar substitute to be 0.9 g allulose/kg·BW for regular ingestion. In other words, the maximum single dose of D-allulose for adults with a mean body weight of 60 kg was 24 g, and up to 54 g can be consumed daily without any side effects. Considering that Koreans consume 61.4 g of total sugar/person/day and 35.0 g sugar/day in the form of processed foods [24], our results suggest that the maximum intake capacity of D-allulose may allow for its use as a partial replacement of added sugars in the Korean diet.

## Figures and Tables

**Table 1:** Dose of sucrose or D-allulose included in the test drinks for occasional ingestion.

**Dose of D-allulose (g/kg·BW)**	0.1 g	0.2 g	0.3 g	0.4 g	0.5 g
**Amount of D-allulose in 200 mL (g)**	6.36 ± 1.19	12.72 ± 2.37	19.08 ± 3.56	25.43 ± 4.74	31.79 ± 5.93

Mean ± S.D.

**Table 2:** Dose of D-allulose included in the test drinks for regular ingestion.

**Dose of D-allulose (g/kg·BW)**	0.1 g	0.2 g	0.3 g
**Amount of D-allulose consumed in 200 mL (g)**	6.42 ± 1.22	12.84 ± 2.43	19.27 ± 3.65

Mean ± S.D.

**Table 3:** Time schedule D-allulose consumption in test drinks for regular ingestion. (unit: g/kg·BW).

Frequency Time	1st	2nd	3rd	4th	5th	6th	7th	8th
**09:00**	-	-	0.1	0.2	0.2	0.2	0.2	0.2
**12:00**	0.2	0.2	0.2	0.2	0.2	0.2	0.3	0.3
**16:00**	-	-	-	-	0.1	0.2	0.2	0.2
**20:00**	-	0.2	0.2	0.2	0.2	0.2	0.2	0.3
**Total**	0.2	0.4	0.5	0.6	0.7	0.8	0.9	1.0

Frequency: The number of times D-allulose was consumed; Time: daily time schedule in which D-allulose was consumed in test drinks; -: No intake the test drinks.

**Table 4:** Ingredient of provide meal.

Ingredient	Amount
**Carbohydrate**	113 g
**Protein**	29 g
**Fat**	22 g
**Calorie**	765 Kcal

**Table 5:** Characteristics of the subjects included in D-allulose gastrointestinal tolerance test.

Age (years)	Height (cm)	Body Weight (kg)	Fat Mass (kg)	Lean Body Mass (kg)	Body Mass Index (kg/m^2^)
23.67 ± 2.01	169.61 ± 7.96	63.58 ± 11.86	13.88 ± 3.83	49.70 ± 9.49	21.91 ± 2.54

Mean ± S.D.

**Table 6:** GI symptoms in the 24 h following the consumption of test drinks containing D-allulose, for occasional ingestion (number of subjects reporting symptoms, *n* = 29 ^a^).

Tolerance Grade ^b^	D-allulose Dose (per kg·BW)				
	0.1 g	0.2 g	0.3 g	0.4 g	0.5 g
**Nausea**					
**0**	24	26	26	27	25
**1**	5	3	2	2	1
**2**	0	0	1	1	3
**3**	0	0	0	0	0
**Total**	**5**	**3**	**3**	**3**	**4**
**Bloating**					
**0**	25	26	27	25	18
**1**	4	3	2	3	10
**2**	0	0	0	1	1
**3**	0	0	0	0	0
**Total**	**4**	**3**	**2**	**4**	11 *
**Borborygmi**					
**0**	28	28	28	28	25
**1**	1	1	1	1	3
**2**	0	0	0	0	1
**3**	0	0	0	0	0
**Total**	**1**	**1**	**1**	**1**	**4**
**Colic**					
**0**	28	28	28	27	20
**1**	1	1	1	2	5
**2**	0	0	0	0	4
**3**	0	0	0	0	0
**Total**	**1**	**1**	**1**	**2**	9 *
**Flatulence**					
**0**	28	29	29	29	27
**1**	1	0	0	0	2
**2**	0	0	0	0	0
**3**	0	0	0	0	0
**Total**	**1**	**0**	**0**	**0**	**2**
**Headache**					
**0**	25	26	25	25	24
**1**	4	2	4	4	1
**2**	0	1	0	0	4
**3**	0	0	0	0	0
**Total**	**4**	**3**	**4**	**4**	**5**
**Diminished appetite**					
**0**	26	26	27	25	27
**1**	3	3	1	2	1
**2**	0	0	1	2	1
**3**	0	0	0	0	0
**Total**	**3**	**3**	**2**	**4**	**2**
**Diarrhea**					
**0**	25	27	26	27	16
**1**	4	2	3	0	4
**2**	0	0	0	2	5
**3**	0	0	0	0	4
**Total**	**4**	**2**	**3**	**2**	13 **

Abbreviation: GI, gastrointestinal. ^a^ 29 subjects completed the study. In one subject, part of the report on the GI symptoms was incomplete. Accordingly, the analysis was carried out on 29 subjects, where appropriate. ^b^ 0 = Normal (no unusual symptoms), 1 = a slightly higher number of symptoms than usual, 2 = a noticeably higher number of symptoms than usual and 3 = a considerably higher number of symptoms than usual. The GI responses following the consumption of the test drinks were compared by the 2 × 2 contingency table analysis (χ^2^) according to the methods of McNemar’s binomial test. The χ^2^ test was used to estimate the differences in the occurrence of multiple symptoms following the consumption of the test products. * Significant increase in the number of subjects experiencing symptoms, relative to the consumption of 0.5 g/kg BW sucrose, * *p* < 0.05, ** *p* < 0.01.

**Table 7:** GI symptoms in the 24 h following the consumption of the test drinks containing sucrose, for occasional ingestion (number of subjects reporting symptoms, *n* = 29 ^a^).

Tolerance Grade ^b^	Sucrose Dose (per kg·BW)				
	0.1 g	0.2 g	0.3 g	0.4 g	0.5 g
**Nausea**					
**0**	28	28	28	25	28
**1**	1	1	1	2	1
**2**	0	0	0	2	0
**3**	0	0	0	0	0
**Total**	**1**	**1**	**1**	**4**	**1**
**Bloating**					
**0**	27	28	27	24	25
**1**	2	1	2	3	3
**2**	0	0	0	2	1
**3**	0	0	0	0	0
**Total**	**2**	**1**	**2**	**5**	**4**
**Borborygmi**					
**0**	27	27	27	26	28
**1**	2	1	2	2	1
**2**	0	1	0	1	0
**3**	0	0	0	0	0
**Total**	**2**	**2**	**2**	**3**	**1**
**Colic**					
**0**	27	28	27	22	25
**1**	2	1	2	5	3
**2**	0	0	0	2	0
**3**	0	0	0	0	0
**Total**	**2**	**1**	**2**	**7**	**3**
**Flatulence**					
**0**	27	28	28	27	28
**1**	2	1	1	1	1
**2**	0	0	0	1	0
**3**	0	0	0	0	0
**Total**	**2**	**1**	**1**	**2**	**1**
**Headache**					
**0**	27	29	27	25	26
**1**	2	0	2	2	1
**2**	0	0	0	1	2
**3**	0	0	0	1	0
**Total**	**2**	**0**	**2**	**4**	**3**
**Diminished appetite**					
**0**	26	26	28	25	28
**1**	3	3	1	3	1
**2**	0	0	0	1	0
**3**	0	0	0	0	0
**Total**	**3**	**3**	**1**	**4**	**1**
**Diarrhea**					
**0**	27	28	26	20	25
**1**	2	1	2	5	2
**2**	0	0	1	4	2
**3**	0	0	0	0	0
**Total**	**2**	**1**	**3**	**9**	**4**

Abbreviation: GI, gastrointestinal. ^a^ 29 subjects completed the study. In one subject, part of the report on the GI symptoms was incomplete. Accordingly, the analysis was carried out on 29 subjects, where appropriate. ^b^ 0 = Normal (no unusual symptoms), 1 = a slightly higher number of symptoms than usual, 2 = a noticeably higher number of symptoms than usual, and 3 = a considerably higher number of symptoms than usual.

**Table 8:** Characteristics of subjects included in the D-allulose gastrointestinal tolerance test (*n* = 19).

Age (Years)	Height (cm)	Body Weight (kg)	Fat Mass (kg)	Lean Body Mass (kg)	Body Mass Index (kg/m^2^)
23.84 ± 2.23	170.00 ± 7.77	64.22 ± 12.17	47.62 ± 8.55	50.38 ± 9.54	21.78 ± 2.66

Mean ± S.D.

**Table 9:** GI symptoms in the 24 h following the consumption of the test drinks containing D-allulose, for regular ingestion (number of subjects reporting symptoms, *n* = 19 ^a^).

Tolerance Grade ^b^	Frequency							
	1st	2nd	3rd	4th	5th	6th	7th	8th
**Nausea**								
**0**	18	17	17	17	17	14	17	14
**1**	1	2	2	1	2	4	0	3
**2**	0	0	0	1	0	1	2	1
**3**	0	0	0	0	0	0	0	1
**Total**	**1**	**2**	**2**	**2**	**2**	**5**	**2**	**5**
**Bloating**								
**0**	16	16	15	14	12	14	12	11
**1**	1	2	2	4	6	4	4	7
**2**	2	1	2	1	1	0	3	1
**3**	0	0	0	0	0	1	0	0
**Total**	**3**	**3**	**4**	**5**	**7**	**5**	**7**	**8**
**Borborygmi**								
**0**	16	14	16	15	15	13	12	11
**1**	0	3	2	2	3	4	4	4
**2**	3	2	1	2	1	2	3	4
**3**	0	0	0	0	0	0	0	0
**Total**	**3**	**5**	**3**	**4**	**4**	**6**	**7**	**8**
**Colic**								
**0**	16	17	15	17	13	16	12	13
**1**	2	0	4	2	6	2	3	3
**2**	1	2	0	0	0	1	4	2
**3**	0	0	0	0	0	0	0	1
**Total**	**3**	**2**	**4**	**2**	**6**	**3**	**7**	**6**
**Flatulence**								
**0**	16	16	17	17	17	15	14	16
**1**	3	3	2	2	2	4	4	3
**2**	0	0	0	0	0	0	1	0
**3**	0	0	0	0	0	0	0	0
**Total**	**3**	**3**	**2**	**2**	**2**	**4**	**5**	**3**
**Headache**								
**0**	17	16	16	15	16	15	17	17
**1**	1	2	3	4	2	4	0	1
**2**	1	1	0	0	1	0	2	0
**3**	0	0	0	0	0	0	0	1
**Total**	**2**	**3**	**3**	**4**	**3**	**4**	**2**	**2**
**Diminished appetite**								
**0**	18	17	18	16	18	17	18	17
**1**	0	2	1	2	1	2	0	1
**2**	1	0	0	1	0	0	1	0
**3**	0	0	0	0	0	0	0	1
**Total**	**1**	**2**	**1**	**3**	**1**	**2**	**1**	**2**
**Diarrhea**								
**0**	18	18	18	16	17	15	17	16
**1**	0	0	0	2	2	4	1	1
**2**	1	1	1	1	0	0	1	0
**3**	0	0	0	0	0	0	0	2
**Total**	**1**	**1**	**1**	**3**	**2**	**4**	**2**	**3**

Abbreviation: GI, gastrointestinal. ^a^ 19 subjects completed the study. In one subject, part of the report on GI symptoms was incomplete. Accordingly, the analysis was carried out on 19 subjects where appropriate. ^b^ 0 = Normal (no unusual symptoms), 1 = slightly more symptoms than usual, 2 = Noticeably more symptoms than usual, and 3 = considerably more symptoms than usual.
